# Occupation and maternal mortality in Brazil

**DOI:** 10.11606/s1518-8787.2020054001736

**Published:** 2020-06-19

**Authors:** Ana Isabela Feitosa-Assis, Vilma Sousa Santana

**Affiliations:** I Universidade Federal da Bahia Instituto de Saúde Coletiva Programa de Pós-Graduação em Saúde Coletiva SalvadorBA Brasil Universidade Federal da Bahia. Instituto de Saúde Coletiva. Programa de Pós-Graduação em Saúde Coletiva. Salvador, BA, Brasil; II Universidade Federal da Bahia Instituto de Saúde Coletiva Programa Integrado em Saúde Ambiental e do Trabalhador SalvadorBA Brasil Universidade Federal da Bahia. Instituto de Saúde Coletiva. Programa Integrado em Saúde Ambiental e do Trabalhador (PISAT). Salvador, BA, Brasil

**Keywords:** Maternal Mortality, Maternal Exposure, Occupational Risks, Occupational Stress, Health Status Disparities

## Abstract

**OBJECTIVE:**

To estimate maternal mortality ratio according to occupation in Brazil.

**METHODS:**

This is a mortality study conducted with national data from the Mortality Information System (SIM) and the Live Birth Information System (SINASC) in 2015. Maternal mortality ratios were estimated according to the occupation recorded in death certificates, using the Brazilian Classification of Occupation (CBO), version 2002.

**RESULTS:**

A total of 1,738 maternal deaths records were found, corresponding to a maternal mortality ratio of 57.6/100,000 live births. It varied among occupational groups, with higher estimates among service and agricultural workers, particularly for domestic workers (123.2/100,000 live births), followed by general agricultural workers (88.3/100,000 live births). Manicurists and nursing technicians also presented high maternal mortality ratio. Maternal occupation was not reported in 17.0% of SIM registers and in 13.2% of SINASC data. Inconsistent records of occupation were found.“Housewife” prevailed in SIM (35.5%) and SINASC (39.1%).

**CONCLUSIONS:**

Maternal mortality ratio differs by occupation, suggesting a work contribution, which requires further research focusing occupational risk factors. Socioeconomic factors are closely related to occupation, and their combination with work exposures and the poor access to health services need to be also addressed.

## INTRODUCTION

The death of women in reproductive age, due to problems of pregnancy, childbirth or puerperium, is unacceptable as it is commonly preventable. Although the maternal mortality ratio (MMR) reduction is a global priority and the existing declining time trend, it has remains at high levels worldwide, as for 216/100,000 live births (LB) in 2015^1^. According to the 10th Revision of the International Classification of Diseases (ICD-10)^2^, maternal death corresponds to “death during pregnancy or up to 42 days after the end of pregnancy, regardless of the duration or location of pregnancy, due to any cause related to or aggravated by pregnancy or by measures in relation to it.” Therefore, it does not include deaths from accidental causes. The codes used are from Chapter XV of ICD-10, “Pregnancy, Childbirth and Puerperium” (excluding codes O96 and O97), in addition to those that occurred during the pregnancy-puerperal status, specifically: disease caused by the human immunodeficiency virus (HIV; B20-B24), neoplasia of uncertain or unknown behavior of the placenta (D39.2), hypopituitarism (E23.0), puerperal osteomalacia (M83.0) obstetric tetanus (A34), and mental and behavioral disorders associated with puerperium (F53)^3^.

In a systematic literature review^4^, worldwide, direct causes of maternal death were the most common (73%), particularly hemorrhages (27.1%) and hypertension (14%), while abortion reaches 7.9%. Among the indirect causes, comorbidities predominated, especially HIV infection, corresponding to 5.5%. Factors related to health care, i.e., quality and access, were also analyzed, but with conflicting findings regarding the absence of prenatal care^5^, reduced number of prenatal care visits^6^ or other aspects of the services organization^7,8^. Research on factors associated with maternal death prevails in countries from the European Union and Africa, among other regions. Associated factors were young^8,9^ or old age^6,10^, single status^6,13^, low schooling^5^, rural housing^8^, and belonging to ethnic minorities^12,15^, specifically Black African or Caribbean^12^, non-Western migrants, Suriname and Dutch Caribbean, other foreigners in the Netherlands^9^, and non-natives in Spain^11^. Smoking was also most common among victims of maternal death^14^. In Brazil, maternal mortality ratio decreased from 2001 to 2012^16^. A study has shown the predominance of direct obstetric causes, especially hypertension, hemorrhages, puerperal infections and abortion^10^, and the association of maternal death with limitations in quality and access to health services^10^. Morse et al.^10^ also pointed to the maternal death relation with social inequalities, revealed by the higher risk among the black, those having lower education or low socioeconomic status. In general, the findings related to socioeconomic conditions suggest that poverty, analyzed with distinct variables, is associated with maternal death, and with vulnerable ethnic groups, migration or occupation, which may play the role of mediators or effect modifiers. Interestingly, despite the multiple evidence that exposure to occupational risk agents (such as chemical substances, physical overload and psychostressors, among others) affects maternal health, reproductive outcomes related to work are still poorly studied.

From few studies showing findings for occupation and maternal death, a number presents only proportions of occupations among cases, but no MMR estimates by occupational groups, in addition to large workers’ groups of types of placement in the labor market, which prevents an adequate understanding of the possible role of work for maternal death. In a study from Mexico, no association was found for unemployment and maternal death^6^, or employment and maternal death in the United Kingdom^15^ or France^7^. In Kenya, a study showed no maternal death differences between women having formal or informal employment when compared with the unemployed^5^. In Tanzania, no distinctions were found across occupations in business or agriculture trades or for those holding temporary jobs when compared with the unemployed^13^. However, with data from the United Kingdom, unemployed (mortality Odds ratio, ORM = 2.50; 95%CI 1.18–5.28)^14^ or those having manual jobs (ORM = 2.19; 95%CI 1.03–4.68) had a higher risk of maternal death than those in managerial occupations^12^. In Brazil, only one study reported no association of paid jobs and maternal death in the city of Campinas, São Paulo^17^. Many of these studies were carried out with small number of subjects wich limits conclusions. This study aims at to estimate the maternal mortality ratio specific for occupational groups in Brazil.

## METHODS

This is a descriptive study of maternal mortality in Brazil in the year 2015. Data sources were the Mortality Information System (SIM), composed by death certificates records, used to retrieve all maternal deaths, according to definition presented further. Live births were obtained from the Live Birth Information System (SINASC), corresponding to live birth declaration (LBD) records, both having national coverage. Anonymous individual databases, from SIM and SINASC, have open acess on the internet at the DATASUS. The referent population comprises all mothers who gave live births. The study population were 10 to 49 years of age, the childbearing age range. The study year corresponds to the one taken for the global evaluation of the Millennium Development Goals.

Maternal deaths cases correspond to SIM records having as the underlying cause, ICD-10 codes O00 to O99, excepting O96 (late maternal death), O97 (death from sequelae of direct obstetric cause), and also deaths considered maternal and non-accidental classified in other chapters (ICD-10 A34, F53, M83.0, B20 to B24, D39.2 and E23.0), with positive answers to questions 43 or 44 of the death certificate, indicating that death occurred with the pregnancy-puerperal cycle. The type of maternal death could be direct-obstetric, “caused by obstetric complications during pregnancy, childbirth or puerperium from interventions, omissions, incorrect treatment or a chain of events resulting from any of these causes” (ICD-10 O00.0 to O08.9, O11 to O23.9, O24.4, O26.0 to O92.7, D39.2, E23.0, F53 and M83.0), or indirect, “caused by diseases previous to pregnancy or which developed during this period, not related to direct obstetric causes, but aggravated by the physiological effects of pregnancy” (ICD-10 O10.0 to O10.9, O24.0 to O24.3, O24.9, O25, O98.0 to O99.8, A34 and B20 to B24), in addition to unspecified obstetric deaths, i.e., those coded as O95 ICD-10.

The central descriptive variable is the “main occupation”, registred in death certificates and LBD, which corresponds “to the type of job that the deceased has developed in most of her productive life.”^18^ These two documents indicate that for retirees or unemployed, one must inform the last usual occupation.^18^ There is also guidance to note “student” when “the person only studied, developing no regularly paid activity.”^18^ The occupation is recorded using the Brazilian Classification of Occupations (CBO), which is based on the International Standardized Classification of Occupations (ISCO), under the responsibility of the International Labor Organization (ILO).

In CBO, occupations are distributed, classified, coded and named in a hierarchical structure, composed of great groups, main subgroups, subgroups, families and occupations (job titles), according to the number of digits used in the corresponding codes. The main occupation, registered in SIM and SINASC with CBO codes (version 2002), was analyzed according to great groups: 1) members of the armed forces, police and military firefighters; 2) senior members of public authorities, leaders of public interest organizations and companies, managers; 3) science and arts professionals; 4) mid-level technicians; 5) administrative service workers; 6) service workers, retail sellers from stores and supermarkets; 7) agricultural, forestry and fishing workers; 8) workers of industrial goods and services production; 9) workers in repair and maintenance services. In addition, although absent in CBO and conceptually inconsistent the following records were separately analyzed: 1) student; 2) housewife; 3) retired/pensioner; 4) unemployed. Moreover, when greater disaggregation was required, occupations were analyzed with codes having more than one digit.

Other descriptive variables were: age in years categorized as age groups (10–14, 15–19, 20–34, 35 and older), race/skin color (white, black, brown, others), marital status (single, married, consensual union, others, ignored), years of schooling (none, 1–3, 4–7, 8–11, 12 and more, ignored), state (Rondônia, Acre, Amazonas, Roraima, Para, Amapá, Tocantins, Maranhão, Piauí, Ceará, Rio Grande do Norte, Paraíba, Paraíba Pernambuco, Alagoas, Sergipe, Bahia, Minas Gerais, Espírito Santo, Rio de Janeiro, São Paulo, Paraná, Santa Catarina, Rio Grande do Sul, Mato Grosso do Sul, Mato Grosso, Goiás) and the Federal District, and country region (North, Northeast, Southeast, Midwest and South).

The maternal mortality ratio (MMR) corresponds to the division between the number of maternal deaths and the number of live births multiplied by 100,000 over a time unit. The overall and specific MMR were estimated by categories of the each descriptive variable. Based on the stratum-specific MMR, MMR ratios (MMR-R) were estimated for great groups of occupations, main subgroups and occupations, using as referent categories, science and arts professionals, and for the remaining, social and human sciences professionals, respectively. Diagnoses of the underlying cause of death, coded by the ICD-10, were analyzed using up to three digits.

Analyses were carried out with the Statistical Analysis Software, version 9.4. We also used Microsoft Office Excel, version 2007, worksheets for exploratory data analysis, and to setup tables. Because these are all secondary data retrieved from open access information systems, of universal coverage, it is not required approval by the National Commission of Ethics in Research (CONEP), Resolution No. 510, April 7, 2016.

## RESULTS

In Brazil, in the year 2015, 1,738 maternal deaths and 3,017,203 live births were registered, corresponding to MMR=57.6 deaths per 100,000 LB. From the total, 17.0% death records from SIM and 13.2% from the mothers of live births database (SINASC) have no maternal occupation data. Table 1 shows that most maternal deaths were from the age group of 20 - 34 years (61.5%) had 8 - 11 years of schooling (40.0%), were single (47.5%) and have brown-skin color (53.9%).

**Table 1:** Distribution of maternal deaths and live births by maternal sociodemographic variables in Brazil, 2015.

Sociodemographic characteristics	Maternal deaths		Mothers of live births	
	
	N	%	N	%
Age group in years				
10–14	13	0.7	26,700	0.9
15–19	222	12.8	520,864	17.2
20–34	1,068	61.5	2,081,723	69.0
35 and older	435	25.0	387,916	12.9
Years of schooling				
None	39	2.4	16,683	0.6
1–3	183	11.3	84,278	2.8
4–7	430	26.5	561,506	18.8
8–11	648	40.0	1,755,605	58.8
12 or more	175	10.8	553,180	18.5
Ignored	145	9.0	16,620	0.5
Marital status				
Single	783	47.5	1,246,029	41.6
Married	485	29.4	990,620	33.0
Consensual union	268	16.3	710,362	23.7
Others	53	3.2	37,880	1.3
Ignored	60	3.6	12,231	0.4
Race/skin color				
White	559	33.3	1,062,962	37.1
Black	176	10.5	149,906	5.2
Brown	903	53.9	1,616,650	56.5
Others	38	2.3	33,044	1.2

Source: Mortality Information System (SIM) and Live Birth Information System (SINASC).

Note: Totals differ due to missing data.

Women living in the São Paulo state (17.9%) and the Southeast region (36.3%) prevailed among maternal deaths. Direct obstetric causes were the most common (66.5%), specifically eclampsia (9.4%), postpartum hemorrhage (7.3%), gestational hypertension (6.9%), obstetric embolism (4.0%), abnormalities of uterine contraction (3.8%), puerperal infection (3.6%), postpartum complications (2.9%), placental abruption (2.8%), other complications of labor and childbirth (2.2%), genitourinary tract infections (2.1%), ectopic pregnancy (2.0%) and abortion (2.0%). Infectious and parasitic maternal diseases contribute most for indirect causes (2.5%) and other maternal diseases that complicate pregnancy, childbirth and puerperium (24.3%). Obstetric deaths of unspecified cause corresponded to 2.5% of the total. Data not presented.

Table 2 shows that the highest MMR were estimated for the great occupational groups of service workers, retail sellers from stores and supermarkets (72.6/100,000 LB) and agricultural, forestry and fishing workers (61.9/100,000 LB); and minors MMR for science and arts professionals (30.0/100,000 LB) and administrative service workers (43.2/100,000 LB). The first two occupational groups mentioned had R MMR=2.4 and 2.1, a maternal death risk over twice higher than the referent group. The occupation data registered as “housewife” was the most frequent in both, SIM (35.6%) and SINASC (39.1%). The corresponding MMR for “housewives” was 52.4/100,000 LB. A total 31 cases of maternal deaths had occupation recorded as ignored.

**Table 2:** Distribution of maternal deaths (SIM) and live births (SINASC), maternal mortality ratio (MMR) and ratio of maternal mortality ratio (R MMR) specific to great groups of maternal occupation. Brazil, 2015.

Great groups (BCO 2002)	Maternal deaths		Mothers of live births		MMR (per 100,000 live births)	R MMR
	
	N	%	N	%		
[2] Science and arts professionals^a^	71	4.1	236,611	7.8	30.0	1.0 (referent)
[5] Service workersretail sellers from stores and supermarkets	209	12.0	287,787	9.5	72.6	2.4
[6] Agricultural, forestry and fishing workers	179	10.3	289,131	9.6	61.9	2.1
[4] Administrative service workers	89	5.1	206,175	6.8	43.2	1.4
[3] Mid-level technicians	58	3.3	117,282	3.9	49.5	1.7
[7-8] Workers of industrial goods and services production	37	2.1	71,927	2.4	51.4	1.7
[1] Senior members of the public authorities, leaders of public interest organizations and companies, managers	27	1.6	53,933	1.8	50.1	1.7
Housewifeb	618	35.6	1,180,364	39.1	52.4	1.7
Student^b^	101	5.8	150,413	5.0	67.1	2.2
Others	22	1.3	18,083	0.6	-	-
Ignored	31	1.8	7,493	0.3	-	-
Missing	296	17.0	398,004	13.2	-	-
Total	1,738	100.0	3,017,203	100.0	57.6	-

CBO 2002: Brazilian Classification of Occupations 2002:

Source: Mortality Information System (SIM) and Live Birth Information System (SINASC).

^a^ Main subgroup of maternal occupation used as referent to estimate the R MMR.

^b^ Categories that, although present in the information systems employed in the study, they are not occupations classified in the CBO 2002.

For cases with consistent occupation data, estimates of MMR and R MMR are presented according to the main subgroups of maternal occupations (Table 3). The services workers showed the highest MMR, 79.4/100,000 LB, followed by agricultural workers (68.0/100,000 LB) and medium-level technicians of biology, biochemistry, health and related sciences (67.8/100,000 LB). Domestic workers had the highest MMR (123.2/100,000 LB), with R MMR almost four times higher than the referent group, as shown in Table 3. Generally, agricultural workers also presented high values of MMR (88.3/100.00 LB), who had R MMR more than twice as high as the referent, as well as manicurists (84.7/100,000 LB), sales representatives (71.4/100,000 LB) and nursing technicians (65.1/100,000 LB).

**Table 3:** Maternal mortality ratio (MMR) and ratio of maternal mortality ratio (R MMR) by main subgroups and maternal occupations. Brazil, 2015.

Main subgroups and occupations (CBO 2002)	MMR (per 100,000 live births)	R MMR
[2] Science and arts professionals		
[23] Social and human sciences professionalsa	26.2	1.0 (referent)
[25] Teaching professionals	45.1	1.7
[22] Biological sciences, health and related professionals	26.6	1.0
[5] Service workers, retail sellers from stores and supermarkets		
[51] Service workers	79.4	3.0
[5121-05] Domestic worker	123.2	4.7
[5161-20] Manicurist	84.7	3.2
[52] Sellers and trade services providers	60.2	2.3
[5211-10] Retail Seller	53.5	2.0
[6] Agricultural, forestry and fishing workers		
[62] Agricultural workers	68.0	2.6
[6210-05] General agricultural worker	88.3	3.4
[6220-20] Seasonal farm worker	59.0	2.3
[61] Producers in agricultural exploitation	40.0	1.5
[6120-05] Agricultural producer	45.1	1.7
[4] Administrative service workers		
[41] Clerks	44.7	1.7
[4110-10] Administrative assistant	33.5	1.3
[42] Customer service workers	41.6	1.6
[4211-25] Cash Operator	38.0	1.5
[3] Mid-level technicians		
[32] Mid-level technicians of biology, biochemistry, health and related sciences	67.8	2.6
[3222-05] Nursing technician	65.1	2.5
[35] Mid-level technicians in administrative sciences	42.7	1.6
[3547-05] Sales Representative	71.4	2.7
[7-8] Workers of industrial goods and services production		
[76] Workers in the textile, tanning, clothing and graphic arts industries	54.4	2.1
[1] Senior members of the public authorities, leaders of public interest organizations and companies, managers		
[14] Managers	56.3	2.1

CBO 2002: Brazilian Classification of Occupations 2002:

Source: Mortality Information System (SIM) and Live Birth Information System (SINASC).

^a^ main subgroup of maternal occupation used as a reference to estimate the R MMR.

The case series analysis showed that among the maternal deaths from the main subgroup of service workers, domestic services, in general, prevailed (45.3%), followed by beauty and personal care services (20.3%), and hospitality and food (13.5%). In the main subgroup of mid-level technicians of biology, biochemistry, health and related sciences, all maternal deaths were technicians from human health sciences. Among the maternal deaths records, the most common specific occupations (CBO six-digit codes) were: general agricultural worker (5.0%), seasonal farm worker (3.4%), maid (2.9%), retail seller (1.7%), cash operator (1.1%), agricultural producer (1.0%), administrative assistant (1.0%), sales representative (1.0%), nursing technician (0.9%) and manicurist (0.9%). Data not presented.

## DISCUSSION

In Brazil, in 2015, maternal mortality ratio varied among occupational groups. The highest maternal death risk was estimated among service and agricultural workers, suggesting that work can contribute to the occurrence of these deaths occurrence, caused by work-related risks factors, or indirectly by low social status determined by job type. Domestic workers, who comprise the main subgroup of service workers, had the highest maternal death risk, as well as manicurists, from this same occupational subgroup. In the 2nd. rank of maternal mortality ratio we found general agricultural workers. Mid-level technicians of biology, biochemistry, health and related sciences hold estimates above the average national RMM, particularly nursing technicians. Distinctively, the lowest RMM estimate corresponds to great groups of science and arts professionals and administrative service workers. Among the direct obstetric causes of maternal deaths, eclampsia, postpartum hemorrhage and gestational hypertension prevailed; specific indirect causes were diseases of the mother that complicated pregnancy, childbirth and puerperium. Most victims were young, had brown skin colour, were single and from the low years of schooling group. Missing data of maternal occupation were observed in SIM and SINASC, as well as data recorded as ignored. Inconsistent registers of occupation, such as “housewife,” absent in CBO, were the most common among maternal deaths and mothers of live birth records.

In this study, the estimated raw MMR, not adjusted by Luizaga et al.^19^ proposed factors, was 60% higher than 35.0/100,000 LB, the target defined in the Millennium Development Goals; it is also far higher than the Brazilian purpose of reaching 30.0/100,000 LB by 2030^a^ under the Sustainable Development Goals agenda. The several strategies adopted in the country, mainly based on the expansion of obstetric care coverage and better training of operational staff did not seem to be fully implemented or effective.

Women’s occupation directly indicates their working conditions, and common jobs tasks indicates homogeneous groups for occupational risk factors. In addition, work-related risk factors could be mediator of socioeconomic status, commonly determined by the occupation level and job tasks. The literature reports that, jobs having high physical demands are associated with adverse pregnancy outcomes, such as spontaneous abortion^20^ or preterm delivery, hypertension or pre-eclampsia^21^ all of them correlated to maternal death. However, studies on direct occupational causes of maternal death are scarce. Occupation was a mediator variable in the McCarthy and Maine^22^ report, who also observed an association of low social status and maternal death.

The higher estimates of MMR found in the great occupational groups of service workers, retail sellers from stores and supermarkets, and agricultural, forestry and fishing workers, suggest a combination role of socioeconomic inequalities and presumable occupational exposures affecting maternal health. Our findings are consistent with those found in the United Kingdom higher risk of maternal death in manual occupations than others^12^. In this investigation, the highest risk of maternal death was estimated among service workers, most (45.3%) domestic workers. They had a risk of maternal death almost four times higher than the referent group. Domestic workers are known for long working hours, commonly above eight hours a day and more than five days a week, in more than one workplace, although they have low wages^23^. A large study on the prevalence of occupational risk factors in pregnancy from Spain found more than 20% for orthostatism, lifting load above 5 kg, intense work rhythms, continuous attention, repetitive and monotonous tasks, work-related stress, lack of support from colleagues and supervisors, social isolation, excessive noise, extreme temperatures and humidity, electromagnetic fields and other physical risk agents^24^. Moreover, in the same study, expressive proportions of pregnant women were exposed to solvents, lead, pesticides, cleaning products, other chemical agents and biological agents^24^. Among domestic workers, physical overload and a few pauses for resting are common, or exposure to chemicals used in cleaning products, such as solvents, associated with increased risk for miscarriage^25^. These factors may contribute to adverse effects on maternal health, both combined or separately, contributing to the higher relative mortality reported for women from service jobs, especially those employed in domestic services.

The occupation subgroup of services also covers beauty and personal care jobs, particularly manicurists, who had higher MMR (84.7/100,000 LB) than the national average. Manicurists manipulate sharp and cutting tools and injuries may expose workers to blood and other materials contaminated by biological agents, such as hepatitis virus, among others. About 10% of the manicurists and pedicurists who undergone serum lab exams tested positive for hepatitis B virus (HBV) and hepatitis C virus (HCV), as reported by Oliveira and Focaccia^26^, with data from São Paulo. Manicurists, hairdressers and beauticians, together are also potentially exposed to chemicals such as epoxys or resins and solvents, known to affect maternal health, while formaldehyde is associated with spontaneous abortion, pulmonary edema or pneumonia, complicating pregnancy, childbirth and puerperium^25^.

In addition to the occupational groups of services, general agricultural workers had a risk of maternal death more than twice as higher as the referent group. A study^8^ found that living in rural areas, common among farm workers, was associated with maternal death. Farming jobs are characterized by high physical demands in almost all tasks performed, in addition to exposure to excessive heat^27^, contact with pesticides^28^ – many of which are endocrine disruptors^29^ – materials and biological agents^30,31^ and animal injuries^31^, among other factors that affect health. Little is known about the working conditions of farm worker women in Brazil, but an important feature is the “naturalization” of occupation, commonly considered only as “help” and not a true job. Therefore, women who work in agriculture, are not likely to be recognized as farm worker, being informed about occupational risks they face and how to prevention them, or whether exposed how they will compromise their health^30,31^. Access to adequate medical care, such as prenatal care, is commonly lower in rural areas^32^, where precarious quality and provision of health services also prevail, which deserves further specific studies.

Another occupational group with MMR higher than the referent group was the mid-level technicians of biology, biochemistry, health and related sciences (67.8/100,000 LB), which comprises only workers from human health sciences. Nursing technicians had MMR of 65.1/100,000 LB, more than the double the comparison group, suggestive of underlying occupational factors. Health professionals, such as nursing technicians, are commonly exposed to high risk of being in contact to biological materials, including infectious agents that cause maternal death, such as HIV infection reported by Tlou et al.^33^ with odds ratio mortality of 2.5 (95%CI 1.5–4.2). Other communicable diseases such as measles, rubella, chickenpox, tuberculosis, pertussis, meningitis, including also infections caused by influenza virus, cytomegalovirus, HBV, HCV and parvovirus B19^25^ may be indirectly associated with maternal death. Other relevant occupational risk factors are long worktime, emotional and physical stress, exposure to anesthetic gases and radiation^34^associated with reproductive effects. Cesarean section, which is associated with hemorrhages, one of the most common causes of maternal death, is more frequent in nurses than in other workers^35^.

However, most studies that addressed the analysis of employment or occupation and type of work did not find differences in the risk of maternal death when compared with referent groups^5^, consistently with the findings of Cecatti et al.^17^. Conclusions are limited, because studies are from very distinct contexts, notably in culture and socioeconomic background, or type of occupations among other. Notably, most studies were conducted with very small samples and restricted statistical power.

The results of this study show occupation-related variation in maternal mortality ratio, suggesting that, in addition to the social determinants already known, working conditions may be relevant information for the prevention of this public health problem. SIM data are limited in scope for not allowing analyses that include factors known to be associated with maternal death, such as socioeconomic status, access, conditions and quality of prenatal care, or even occupation group, for adjustment or stratification. In this sense, our conclusions should be considered with caution. Among the methodological limits, under-registration of maternal death are likely to occur. This was shown by Luizaga et al.^19^: data on under-enumeration of maternal deaths from SIM, which although reducing, still reached 21.4%. Another relevant methodological limit is the poor quality and missing occupation data from SIM and SINASC. Missing data on occupation were more common in SIM than in SINASC, which may be a consequence of distinct informants used in each data source, revealing differences when these information systems are compared. In SIM, the occupation can be obtained from the deceased identification papers, available to the attestant, or information from someone close to them, while for SINASC the informant is the mother herself. Moreover, the registered occupation may not be the true, considering the large number of CBO codes to be consulted, limiting the registration, which support the need for better training for the attesting staff. This could be see in the large number of inconsistencies, such as the high proportion of “housewives” (35.5%).Although it is an occupation used for social security purposes, does not integrate the CBO. In Brazil, maternal occupation was also no longer recorded in 14.8% in live birth declarations^36^. Considering that the under-reporting of maternal deaths or missing occupation data are plausible and unequal across country regions and federation units, or occupations, biases may occur limiting the national estimates presented. The use of distinct data sources, SIM and SINASC, which have operational differences in data completion and flow, may lead to bias, although both are housed in DATASUS and subject to similar control and retrieving mechanisms. Moreover, although the investigation of maternal deaths has been advancing across the country, it has been implemented heterogeneously; however, it did not occur in the occupation field record.

Research on occupational determinants of maternal death requires investments, especially for studies conducted with detailed primary data on working and socioeconomic conditions, among others. The findings of this study may contribute to a better visibility of the problem and alert to new contributions aimed at the most vulnerable occupational groups. Efficient strategies, in this sense, should integrate lawsuits and supervision, regulations and implementation of special programs, in addition to legislation aimed to guarantee human and labor rights.

## Footnotes

a Ministério da Saúde (BR). Ministério da Saúde investe na redução da mortalidade materna. Brasília, DF: Agência Saúde; 28 maio 2018 [cited 2018 Dec 21]. Available from: http://portalms.saude.gov.br/noticias/agencia-saude/43325-ministerio-da-saude-investe-na-reducao-da-mortalidade-materna
