# Supplementary material for: Occupation and maternal mortality in Brazil
Source: Rev Saude Publica. 2020 Jun 19;54:64. doi: 10.11606/s1518-8787.2020054001736 (PMC7304881; doi:10.11606/s1518-8787.2020054001736)
Supplement: Translation

# Translation: Ocupação e mortalidade materna

**Authors:** Ana Isabela Feitosa-Assis, Vilma Sousa Santana

**Affiliations:** I Brasil Universidade Federal da Bahia. Instituto de Saúde Coletiva. Programa de Pós-Graduação em Saúde Coletiva. Salvador, BA, Brasil; II Brasil Universidade Federal da Bahia. Instituto de Saúde Coletiva. Programa Integrado em Saúde Ambiental e do Trabalhador (PISAT). Salvador, BA, Brasil

**Keywords:** Mortalidade Materna, Exposição Materna, Riscos Ocupacionais, Estresse Ocupacional, Desigualdades em Saúde

## Abstract

**OBJETIVO:**

Estimar a razão de mortalidade materna segundo a ocupação no Brasil.

**MÉTODOS:**

Trata-se de estudo de mortalidade realizado com dados nacionais do Sistema de Informação sobre Mortalidade (SIM) e do Sistema de Informação sobre Nascidos Vivos (Sinasc) no ano de 2015. Foram estimadas as razões de mortalidade materna de acordo com a ocupação registrada em declarações de óbito, utilizando a Classificação Brasileira de Ocupações, versão 2002.

**RESULTADOS:**

Foram encontrados 1.738 registros de óbitos maternos, correspondendo a uma razão de mortalidade materna de 57,6/100.000 nascidos vivos. Esse índice variou entre as categorias ocupacionais, sendo maior entre as trabalhadoras dos serviços e da agropecuária, particularmente para as empregadas domésticas (123,2/100.000 nascidos vivos), seguidas pelas trabalhadoras agropecuárias em geral (88,3/100.000 nascidos vivos). Também apresentaram elevada razão de mortalidade materna as manicures e técnicas de enfermagem. A ocupação materna não foi informada em 17,0% dos casos do SIM e em 13,2% do Sinasc. Foram encontrados registros inconsistentes, como “dona de casa”, o mais frequente no SIM (35,5%) e no Sinasc (39,1%).

**CONCLUSÕES:**

A razão de mortalidade materna difere de acordo com a ocupação, sugerindo uma contribuição do trabalho, o que requer pesquisas adicionais para identificar os fatores de risco ocupacionais. Fatores socioeconômicos estão intimamente relacionados à ocupação, e sua combinação com exposições no trabalho e acesso a serviços de saúde precisa ser abordada.

## INTRODUÇÃO

A morte de mulheres em idade reprodutiva, decorrente de problemas da gravidez, parto ou puerpério, é inaceitável por ser comumente evitável. Apesar de a redução da razão de mortalidade materna (RMM) ser prioridade global e haver uma tendência geral de queda, vem se mantendo ainda em níveis elevados mundialmente, como o de 216/100.000 nascidos vivos (NV) em 2015^1^. Segundo a 10ª Revisão da Classificação Internacional de Doenças (CID-10)^2^, morte materna corresponde à “morte durante a gestação ou até 42 dias após o término da gestação, independente da duração ou da localização da gravidez, devida a qualquer causa relacionada com ou agravada pela gravidez ou por medidas em relação a ela”. Portanto, não inclui as ocorridas por causas acidentais. Os códigos utilizados são do Capítulo XV da CID-10, “Gravidez, Parto e Puerpério” (excluídos os códigos O96 e O97), além das que comprovadamente ocorreram durante o estado gravídico-puerperal, especificamente: doença causada pelo vírus da imunodeficiência humana (HIV; B20-B24), neoplasia de comportamento incerto ou desconhecido da placenta (D39.2), hipopituitarismo (E23.0), osteomalácia puerperal (M83.0), tétano obstétrico (A34) e transtornos mentais e comportamentais associados ao puerpério (F53)^3^.

Em uma revisão sistemática de literatura recente^4^, no mundo, as causas diretas da morte materna foram as mais comuns (73%), entre as quais destacaram-se as hemorragias (27,1%) e doenças hipertensivas (14%), enquanto o aborto representou 7,9%. Entre as causas indiretas predominaram as comorbidades, especialmente a infecção pelo HIV, que contribuiu com 5,5%. Analisaram-se também fatores relativos ao cuidado, qualidade e acesso, com achados controversos em relação à ausência de atendimento pré-natal^5^, número reduzido de consultas^6^ ou outros aspectos da organização dos serviços^7,8^. Pesquisas sobre fatores associados à morte materna se concentraram em países da União Europeia e África, entre outras regiões. Fatores associados foram a idade jovem^8,9^ e mais avançada^6,10^, o estado civil solteira^6,13^, a baixa escolaridade^5^, residência em área rural^8^ e pertencimento a minorias étnicas^12,15^, especificamente negros africanos ou caribenhos^12^, migrantes não ocidentais, do Suriname e Caribe holandês, demais estrangeiros na Holanda^9^ e não nativos na Espanha^11^. O hábito de fumar também foi mais comum entre as mulheres vítimas de morte materna^14^. No Brasil, a mortalidade materna apresentou tendência de queda entre 2001 e 2012^16^. Um estudo demonstrou a predominância de causas obstétricas diretas, especialmente as doenças hipertensivas, hemorragias, infecções puerperais e o aborto^10^, e associação da morte materna com limitações da qualidade e do acesso aos serviços de saúde^10^. Morse et al.^10^ também apontaram para a relação com as desigualdades sociais, evidente no risco maior de morte materna para mulheres negras, de menor escolaridade ou de baixo nível socioeconômico. Em geral, os achados relativos às condições socioeconômicas sugerem que a pobreza, analisada com diferentes variáveis, se associa à morte materna, bem como a grupos étnicos vulneráveis, a migração ou mesmo a ocupação, que podem ser mediadoras ou modificadoras de efeito. Interessante é que, a despeito das múltiplas evidências de que a exposição a agentes de risco ocupacionais (como substâncias químicas, sobrecarga física e psicoestressores, entre outros) afeta a saúde materna, os desfechos reprodutivos relacionados ao trabalho são ainda pouco estudados.

Entre os raros estudos que mostram achados ocupacionais para a morte materna, boa parte limita-se à apresentação de proporções de ocupações entre os casos mas não mostram estimativas da RMM por grupos de ocupação, ou realizou apenas comparações de grandes grupos relativos à inserção no mercado de trabalho, o que impede a adequada compreensão sobre o possível papel do trabalho na morte materna. Em um estudo realizado no México, não houve associação entre desemprego e morte materna^6^, assim como não houve associação entre emprego e a morte materna no Reino Unido^15^ e na França^7^. No Quênia, também não foram encontradas diferenças na morte materna entre mulheres com emprego formal e informal quando comparadas às desempregadas^5^, nem na Tanzânia, ao serem analisados grupos ocupacionais de negócios, agricultura e com vínculos temporários em comparação ao grupo de desempregadas^13^. Todavia, com dados do Reino Unido, verificou-se que mulheres desempregadas (Odds ratio da mortalidade, MOR = 2,50; IC95% 1,18–5,28)^14^ ou em atividades de trabalho manual (MOR = 2,19; IC95% 1,03–4,68) apresentavam maior risco de morte materna quando comparadas às de ocupações administrativas^12^. No Brasil, apenas um estudo apresentou achados de não associação com o emprego remunerado no município de Campinas, São Paulo^17^. Muitos desses estudos foram conduzidos com amostras pequenas que limitam as conclusões. Neste estudo, pretende-se estimar a razão de mortalidade materna por grupo ocupacional no Brasil.

## MÉTODOS

Este é um estudo descritivo da mortalidade materna no Brasil para o ano de 2015. A fonte de dados foi o Sistema de Informação sobre Mortalidade (SIM), que congrega registros de declarações de óbito (DO), dos quais foram selecionados os óbitos maternos, conforme definição adiante. Dados sobre nascidos vivos foram obtidos do Sistema de Informação sobre Nascidos Vivos (Sinasc), dos registros de declaração de nascido vivo (DNV), ambos para todo o território nacional. Bases de dados individuados, anônimos, do SIM e Sinasc estão disponíveis publicamente no Datasus. A população de referência compreende todas as mães de nascidos vivos. A população de estudo tinha idade entre 10 e 49 anos (idade fértil). O ano de estudo foi escolhido por ser o da avaliação global dos Objetivos de Desenvolvimento do Milênio.

Casos de morte por causa materna correspondem a registros no SIM classificados segundo o Capítulo XV da CID-10, códigos de O00 a O99, com exceção dos códigos O96 (morte materna tardia) e O97 (morte por sequela de causa obstétrica direta), e também as mortes consideradas maternas e não acidentais classificadas em outros capítulos (CID-10 A34, F53, M83.0, B20 a B24, D39.2 e E23.0), com respostas positivas às questões 43 ou 44 da DO, indicando que a morte ocorreu no ciclo gravídico-puerperal. O tipo de morte materna poderia ser obstétrica direta, “que ocorre por complicações obstétricas durante gravidez, parto ou puerpério devido a intervenções, omissões, tratamento incorreto ou a uma cadeia de eventos resultantes de qualquer dessas causas” (CID-10 O00.0 a O08.9, O11 a O23.9, O24.4, O26.0 a O92.7, D39.2, E23.0, F53 e M83.0), ou indireta, “resultante de doenças que existiam antes da gestação ou que se desenvolveram durante esse período, não provocadas por causas obstétricas diretas, mas agravadas pelos efeitos fisiológicos da gravidez” (CID-10 O10.0 a O10.9, O24.0 a O24.3, O24.9, O25, O98.0 a O99.8, A34 e B20 a B24), além de obstétrica não especificada, que abrange óbitos codificados pela CID-10 como O95.

A variável descritora principal é a ocupação habitual, presente na DO e DNV, que corresponde “ao tipo de trabalho que o falecido desenvolveu na maior parte da sua vida produtiva”^18^. Nesses dois documentos indica-se que para aposentadas ou desempregadas deve-se informar a última ocupação habitual^18^. Há também a orientação para anotar “estudante” quando “apenas estudava e não desenvolvia nenhuma atividade regularmente remunerada”^18^. A ocupação é registrada empregando-se a Classificação Brasileira de Ocupações (CBO), que se baseia na *International Standardized Classification of Occupations* (ISCO), de responsabilidade da Organização Internacional do Trabalho (OIT).

Na CBO, as ocupações são distribuídas e classificadas em uma estrutura hierárquica, composta por grandes grupos, subgrupos principais, subgrupos, famílias e ocupações, nominados de acordo com o número de dígitos dos códigos. A ocupação, registrada no SIM e Sinasc com os códigos da CBO (versão 2002), foi analisada segundo grandes grupos: 1) membros das forças armadas, policiais e bombeiros militares; 2) membros superiores do poder público, dirigentes de organizações de interesse público e de empresas, gerentes; 3) profissionais das ciências e das artes; 4) técnicos de nível médio; 5) trabalhadores de serviços administrativos; 6) trabalhadores dos serviços, vendedores do comércio em lojas e mercados; 7) trabalhadores agropecuários, florestais e da pesca; 8) trabalhadores da produção de bens e serviços industriais; 9) trabalhadores em serviços de reparação e manutenção. Além desses, foram considerados os seguintes grupos de “ocupações”, assim denominados, ainda que inconsistentes do ponto de vista conceitual e da CBO: 1) estudante; 2) dona de casa; 3) aposentada/pensionista; 4) desempregada. Ademais, quando necessária maior desagregação, a ocupação foi analisada com códigos de mais de um dígito.

Outras variáveis descritoras são: idade em anos classificada em faixa etária (10–14, 15–19, 20–34, 35 e mais), raça/cor (branca, preta, parda, outras), estado civil (solteira, casada, união consensual, outros, ignorado), escolaridade em anos de estudo (nenhum, 1–3, 4–7, 8–11, 12 e mais, ignorada), procedência por unidade da federação (Rondônia, Acre, Amazonas, Roraima, Para, Amapá, Tocantins, Maranhão, Piauí, Ceará, Rio Grande do Norte, Paraíba, Pernambuco, Alagoas, Sergipe, Bahia, Minas Gerais, Espírito Santo, Rio de Janeiro, São Paulo, Paraná, Santa Catarina, Rio Grande do Sul, Mato Grosso do Sul, Mato Grosso, Goiás, Distrito Federal) e região geográfica (Norte, Nordeste, Sudeste, Centro-Oeste e Sul).

A razão de mortalidade materna (RMM) corresponde à divisão entre o número de mortes maternas e o de nascidos vivos multiplicada por 100 mil. Estimou-se a RMM geral e a específica para as categorias das variáveis descritoras. Posteriormente, calculou-se a partir da RMM específica a razão da RMM (R RMM) para os grandes grupos, subgrupos principais e ocupações, utilizando-se como referência para os primeiros as profissionais das ciências e das artes e para os demais as profissionais das ciências sociais e humanas. Diagnósticos da causa básica de óbito, codificados pela CID-10, foram analisados de acordo com as categorias de três caracteres.

As análises foram realizadas com o programa estatístico *Statistical Analysis Software*, versão 9.4. Também foi utilizado o programa *Microsoft Office Excel*, versão 2007, para tabulação e análise exploratória dos dados, apresentados em forma de tabelas. Por se tratar de estudo com dados secundários extraídos de sistemas de informação de cobertura universal e acesso público, não foi necessária a submissão à Comissão Nacional de Ética em Pesquisa (CONEP), conforme a Resolução nº 510, de 7 de abril de 2016.

## RESULTADOS

No Brasil, em 2015, foram registrados 1.738 óbitos maternos e 3.017.203 nascidos vivos, o que corresponde a uma RMM de 57,6 óbitos a cada 100 mil NV nesse ano. Houve dados faltantes para a ocupação materna em 17,0% dos registros de óbitos no SIM e 13,2% das mães de nascidos vivos no SINASC. Na Tabela 1 observa-se que a maior parte das mortes maternas representava mulheres de 20 a 34 anos de idade (61,5%) e 8 a 11 anos de estudo (40,0%), solteiras (47,5%) e pardas (53,9%).

**Tabela 1:** Distribuição dos óbitos maternos e nascidos vivos por variáveis sociodemográficas maternas no Brasil, 2015.

Variáveis sociodemográficas	Óbitos maternos		Mães de nascidos vivos	
	
	N	%	N	%
Faixa etária em anos				
10–14	13	0,7	26.700	0,9
15–19	222	12,8	520.864	17,2
20–34	1.068	61,5	2.081.723	69,0
35 e mais	435	25,0	387.916	12,9
Anos de estudo				
Nenhum	39	2,4	16.683	0,6
1–3	183	11,3	84.278	2,8
4–7	430	26,5	561.506	18,8
8–11	648	40,0	1.755.605	58,8
12 e mais	175	10,8	553.180	18,5
Ignorado	145	9,0	16.620	0,5
Estado civil				
Solteira	783	47,5	1.246.029	41,6
Casada	485	29,4	990.620	33,0
União consensual	268	16,3	710.362	23,7
Outros	53	3,2	37.880	1,3
Ignorado	60	3,6	12.231	0,4
Raça/cor				
Branca	559	33,3	1.062.962	37,1
Preta	176	10,5	149.906	5,2
Parda	903	53,9	1.616.650	56,5
Outras	38	2,3	33.044	1,2

Fonte: Sistema de Informação sobre Mortalidade (SIM) e Sistema de Informação sobre Nascidos Vivos (Sinasc).

Nota: Totais diferem devido a dados sociodemográficos faltantes.

Mulheres procedentes do estado de São Paulo (17,9%) e da região Sudeste (36,3%) predominaram entre os óbitos maternos. Causas obstétricas diretas foram as mais prevalentes (66,5%), especificamente a eclampsia (9,4%), hemorragia pós-parto (7,3%), hipertensão gestacional (6,9%), embolia de origem obstétrica (4,0%), anormalidades da contração uterina (3,8%), infecção puerperal (3,6%), complicações do puerpério (2,9%), descolamento prematuro de placenta (2,8%), outras complicações do trabalho de parto e do parto (2,2%), infecções do trato geniturinário (2,1%), gravidez ectópica (2,0%) e aborto (2,0%). Predominaram entre as causas indiretas as doenças infecciosas e parasitárias maternas (2,5%) e outras doenças da mãe que complicam a gravidez, o parto e o puerpério (24,3%). Mortes obstétricas de causa não especificada corresponderam a 2,5% do total. Esses dados não foram apresentados.

Na Tabela 2 verifica-se que as maiores RMM foram estimadas para os grandes grupos ocupacionais das trabalhadoras dos serviços, vendedoras do comércio em lojas e mercados (72,6/100.000 NV) e trabalhadoras agropecuárias, florestais e da pesca (61,9/100.000 NV); e os menores para as profissionais das ciências e das artes (30,0/100.000 NV) e trabalhadoras de serviços administrativos (43,2/100.000 NV). Os dois primeiros grupos ocupacionais mencionados tiveram R RMM 2,4 e 2,1 maiores que o grande grupo de referência. Dentre os registros no campo ocupação, “dona de casa” foi o mais frequente no SIM (35,6%) e no Sinasc (39,1%). Sob essa denominação ocupacional, a RMM correspondente foi de 52,4/100.000 NV. Foram 31 casos de mortes maternas com registros de ocupação ignorada.

**Tabela 2:** Distribuição de óbitos maternos (SIM) e nascidos vivos (Sinasc), razão de mortalidade materna (RMM) e razão da razão de mortalidade materna (R RMM) específicas por grandes grupos de ocupação materna. Brasil, 2015.

Grandes grupos (CBO 2002)	Óbitos maternos		Mães de nascidos vivos		RMM (por 100.000 nascidos vivos)	R RMM
	
	N	%	N	%		
[2] Profissionais das ciências e das artes^a^	71	4,1	236.611	7,8	30,0	1,0 (referente)
[5] Trabalhadores dos serviços, vendedores do comércio em lojas e mercados	209	12,0	287.787	9,5	72,6	2,4
[6] Trabalhadores agropecuários, florestais e da pesca	179	10,3	289.131	9,6	61,9	2,1
[4] Trabalhadores de serviços administrativos	89	5,1	206.175	6,8	43,2	1,4
[3] Técnicos de nível médio	58	3,3	117.282	3,9	49,5	1,7
[7-8] Trabalhadores da produção de bens e serviços industriais	37	2,1	71.927	2,4	51,4	1,7
[1] Membros superiores do poder público, dirigentes de organizações de interesse público e de empresas, gerentes	27	1,6	53.933	1,8	50,1	1,7
Dona de casa^b^	618	35,6	1.180.364	39,1	52,4	1,7
Estudante^b^	101	5,8	150.413	5,0	67,1	2,2
Outros	22	1,3	18.083	0,6	-	-
Ignorada	31	1,8	7.493	0,3	-	-
Sem informação	296	17,0	398.004	13,2	-	-
Total	1.738	100,0	3.017.203	100,0	57,6	-

CBO 2002: Classificação Brasileira de Ocupações de 2002

Fonte: Sistema de Informação sobre Mortalidade (SIM) e Sistema de Informação sobre Nascidos Vivos (Sinasc).

^a^ Grande grupo de ocupação materna utilizado como referência para estimar a R RMM.

^b^ Categorias que, embora estejam presentes nos sistemas de informação empregados no estudo, não são ocupações classificadas na CBO 2002.

Para os casos com informações ocupacionais consistentes, apresentam-se estimativas da RMM e R RMM de acordo com subgrupos principais de ocupações maternas (Tabela 3). As trabalhadoras dos serviços apresentaram a maior RMM, 79,4/100.000 NV, seguidas das trabalhadoras classificadas na exploração agropecuária (68,0/100.000 NV) e técnicas de nível médio das ciências biológicas, bioquímicas, da saúde e afins (67,8/100.000 NV). Empregadas em serviços domésticos tiveram a mais alta RMM (123,2/100.000 NV), com R RMM quase quatro vezes maior que o grupo de referência, conforme apresentado na Tabela 3. Também apresentaram valores elevados da RMM as trabalhadoras da agropecuária em geral (88,3/100.00 0NV), que tiveram R RMM mais de duas vezes maior do que o referente, bem como manicures (84,7/100.000 NV), representantes comerciais autônomas (71,4/100.000 NV) e técnicas de enfermagem (65,1/100.000 NV).

**Tabela 3:** Razão de mortalidade materna (RMM) e razão da razão de mortalidade materna (R RMM) específicas por subgrupos principais e ocupações maternas. Brasil, 2015.

Subgrupos principais e ocupações (CBO 2002)	RMM (por 100.000 nascidos vivos)	R RMM
[2] Profissionais das ciências e das artes		
[23] Profissionais das ciências sociais e humanas^a^	26,2	1,0 (referente)
[25] Profissionais do ensino	45,1	1,7
[22] Profissionais das ciências biológicas, da saúde e afins	26,6	1,0
[5] Trabalhadores dos serviços, vendedores do comércio em lojas e mercados		
[51] Trabalhadores dos serviços	79,4	3,0
[5121-05] *Empregado doméstico nos serviços gerai*s	123,2	4,7
[5161-20] *Manicure*	84,7	3,2
[52] Vendedores e prestadores dos serviços do comércio	60,2	2,3
[5211-10] *Vendedor de comércio varejista*	53,5	2,0
[6] Trabalhadores agropecuárias, florestais e da pesca		
[62] Trabalhadores na exploração agropecuária	68,0	2,6
[6210-05] *Trabalhador agropecuário em geral*	88,3	3,4
[6220-20] *Trabalhador volante da agricultura*	59,0	2,3
[61] Produtores na exploração agropecuária	40,0	1,5
[6120-05] *Produtor agrícola polivalente*	45,1	1,7
[4] Trabalhadores de serviços administrativos		
[41] Escriturários	44,7	1,7
[4110-10] *Assistente administrativo*	33,5	1,3
[42] Trabalhadores de atendimento ao público	41,6	1,6
[4211-25] *Operador de caixa*	38,0	1,5
[3] Técnicos de nível médio		
[32] Técnicos de nível médio das ciências biológicas, bioquímicas, da saúde e afins	67,8	2,6
[3222-05] *Técnico de enfermagem*	65,1	2,5
[35] Técnicos de nível médio nas ciências administrativas	42,7	1,6
[3547-05] *Representante comercial autônomo*	71,4	2,7
[7-8] Trabalhadores da produção de bens e serviços industriais		
[76] Trabalhadores nas indústrias têxtil, do curtimento, do vestuário e das artes gráficas	54,4	2,1
[1] Membros superiores do poder público, dirigentes de organizações de interesse público e de empresas, gerentes		
[14] Gerentes	56,3	2,1

CBO 2002: Classificação Brasileira de Ocupações de 2002

Fonte: Sistema de Informação sobre Mortalidade (SIM) e Sistema de Informação sobre Nascidos Vivos (Sinasc).

^a^ Subgrupo principal de ocupação materna utilizado como referência para estimar a R RMM.

A análise da casuística mostrou que, entre as mortes maternas do subgrupo principal de trabalhadoras dos serviços, predominaram as empregadas em serviços domésticos em geral (45,3%), dos serviços de embelezamento e cuidados pessoais (20,3%) e de hotelaria e alimentação (13,5%). No subgrupo principal das técnicas de nível médio das ciências biológicas, bioquímicas, da saúde e afins, 100% dos óbitos maternos foram de técnicas da ciência da saúde humana. Entre os registros de óbitos maternos, as ocupações específicas (com códigos de seis dígitos na CBO) mais frequentes foram: trabalhadora agropecuária em geral (5,0%), trabalhadora volante da agricultura (3,4%), empregada doméstica (2,9%), vendedora de comércio varejista (1,7%), operadora de caixa (1,1%), produtora agrícola polivalente (1,0%), assistente administrativa (1,0%), representante comercial autônoma (1,0%), técnica de enfermagem (0,9%) e manicure (0,9%). Esses dados não foram apresentados.

## DISCUSSÃO

No Brasil, em 2015, a razão de mortalidade materna variou entre as categorias ocupacionais, com as maiores estimativas para as trabalhadoras de serviços e da agropecuária, achados sugestivos de que o trabalho pode influenciar a ocorrência desses óbitos, seja por fatores de riscos relacionados à atividade ocupacional, ou pelas desigualdades sociais determinadas pelo tipo de trabalho. Empregadas domésticas, que integram o subgrupo principal das trabalhadoras de serviços, apresentaram o maior risco de morte materna, como também as manicures deste mesmo subgrupo. Em segundo lugar ficaram as trabalhadoras agropecuárias em geral. Com estimativas acima da média nacional situaram-se as técnicas de nível médio das ciências biológicas, bioquímicas, da saúde e afins, especificamente as técnicas de enfermagem. Distintamente, a menor estimativa corresponde aos grandes grupos das profissionais das ciências e das artes e trabalhadoras de serviços administrativos. Entre as causas obstétricas diretas prevaleceram a eclampsia, hemorragia pós-parto e hipertensão gestacional; entre as indiretas, doenças da mãe que complicaram a gravidez, o parto e o puerpério. A maior parte era de mulheres jovens, pardas, solteiras e de baixa instrução. Dados faltantes de ocupação materna foram observados no SIM e Sinasc, como também o registro como ignorada. Registros inconsistentes da ocupação, como “dona de casa”, não incluídos na CBO, foram os mais frequentes entre os óbitos maternos e mães de nascidos vivos.

Neste estudo, a RMM geral estimada, sem a aplicação de fatores de correção empregada por Luizaga et al.^19^, foi 60% superior à meta de 35,0/100.000 NV definida nos Objetivos de Desenvolvimento do Milênio, como também distante da meta brasileira de 30,0/100.000 NV até 2030^a^ para os Objetivos do Desenvolvimento Sustentável. As diferentes estratégias adotadas no país, sustentadas principalmente na ampliação da cobertura do atendimento em obstetrícia e capacitação do corpo funcional das instituições de saúde, parecem não ter sido implementadas ou efetivas.

A ocupação da mulher indica diretamente as condições de trabalho, ao representar grupos homogêneos de fatores de riscos ocupacionais, além da mediação do *status* socioeconômico comumente determinado pela qualificação do emprego e atividade ocupacional. Na literatura, altas demandas físicas no trabalho se associam a efeitos adversos na gestação, como o aborto espontâneo^20^ ou o parto pré-termo e hipertensão ou pré-eclampsia^21^ envolvidos na morte materna. Estudos sobre causas diretamente ocupacionais da morte materna são escassos. Mas a ocupação foi encontrada como variável de mediação por McCarthy e Maine^22^ que observaram para o efeito da condição social na determinação da morte materna.

As maiores estimativas da RMM apresentadas pelos grandes grupos ocupacionais das trabalhadoras dos serviços, vendedoras do comércio em lojas e mercados e trabalhadoras agropecuárias, florestais e da pesca sugerem desigualdades socioeconômicas e prováveis exposições ocupacionais que afetam a saúde materna. Esses achados são consistentes com os encontrados no Reino Unido de que mulheres que trabalhavam em atividades manuais tiveram maior risco de morte materna que as demais^12^. Nesta investigação, o maior risco de morte materna foi estimado entre trabalhadoras dos serviços, entre as quais grande parte (45,3%) é representada por trabalhadoras dos serviços domésticos em geral. As empregadas domésticas tiveram risco de morte materna quase quatro vezes maior que o grupo de referência. As condições de trabalho desse grupo são conhecidas por extensas jornadas de trabalho, comumente acima de oito horas diárias e mais de cinco dias por semana, em mais de um local de trabalho e com baixos salários^23^. Um amplo estudo sobre a prevalência de fatores de risco ocupacionais na gravidez desenvolvido na Espanha estimou valores acima de 20% para ortostatismo, elevação de carga acima de 5 kg, ritmos intensos de trabalho, exigência de atenção constante, tarefas repetitivas e monótonas, estresse laboral, falta de apoio de colegas e supervisores, isolamento social, ruído excessivo, temperaturas extremas e umidade, campos eletromagnéticos e outros agentes de risco físico^24^. Ademais, nesse mesmo estudo estimaram-se expressivas proporções de gestantes expostas a solventes, chumbo, pesticidas, produtos de limpeza, outros agentes químicos e agentes biológicos^24^. No emprego em serviços domésticos são comuns excessos de carga física e poucas pausas para descanso, como também a exposição a produtos químicos presentes em materiais de limpeza, a exemplo de solventes, que se associam ao aumento de risco para o aborto espontâneo^25^. Combinados ou separadamente, esses fatores podem contribuir para efeitos adversos sobre a saúde materna, concorrendo para a maior mortalidade relativa do grupo de trabalhadoras dos serviços, em especial das empregadas em serviços domésticos.

O subgrupo ocupacional de serviços também abrange atividades de embelezamento e cuidados pessoais, com destaque para as manicures, que apresentaram elevada RMM (84,7/100.000 NV) em relação à média nacional. Manicures manipulam instrumentos e materiais perfurocortantes, e acidentes com lesões podem gerar o contato com sangue e outros materiais contaminados por agentes biológicos causadores de doenças, como as hepatites, entre outras. Cerca de 10% das manicures e pedicures avaliadas tinham sorologia positiva para o vírus da hepatite B (VHB) e vírus da hepatite C (VHC) no estudo de Oliveira e Focaccia^26^, realizado em São Paulo. Juntamente com cabelereiras e esteticistas, essas profissionais também estão potencialmente expostas a produtos químicos, como epóxis ou resinas e solventes, conhecidos por afetarem a saúde materna, enquanto o formaldeído se associa ao aborto espontâneo, edema pulmonar ou pneumonia, complicando a gravidez, o parto e puerpério^25^.

Além dos grupos ocupacionais de serviços, trabalhadoras da agropecuária em geral tiveram risco de morte materna mais de duas vezes maior que as do grupo de referência. Em um estudo^8^ foi encontrada associação de residência na área rural, comum em agricultoras, com a morte materna. Essa ocupação se caracteriza por altas exigências físicas em quase todas as atividades envolvidas, além de exposição ao calor excessivo^27^, contato com agrotóxicos^28^ – muitos dos quais são disruptores endócrinos^29^ –, materiais e agentes biológicos^30,31^ e acidentes com animais^31^, entre outros fatores que afetam a saúde. Pouco se sabe sobre as condições de trabalho de agricultoras no Brasil, mas um aspecto importante é a “naturalização” da ocupação, considerada comumente apenas como “ajuda” e não trabalho de fato. Com isso, mulheres que exercem atividades na agricultura, ao não serem reconhecidas como agricultoras não são informadas sobre os agentes de risco ocupacionais e medidas de prevenção, nem que são expostas a esses fatores que comprometem sua saúde^30,31^. O acesso à assistência médica adequada, como o pré-natal, comumente é menor em áreas rurais^32^, onde prevalecem também a precariedade da qualidade e oferta de serviços de saúde, o que merece estudos específicos.

Um outro grupo ocupacional afetado por RMM em maior nível que o de referência foi o das técnicas de nível médio das ciências biológicas, bioquímicas, da saúde e afins (67,8/100.000 NV), que compreendia apenas trabalhadoras das ciências da saúde humana e afins. Técnicas de enfermagem apresentaram a RMM de 65,1/100.000 NV, mais que o dobro do grupo de comparação, o que sugere fatores ocupacionais envolvidos. Profissionais de saúde, a exemplo das técnicas de enfermagem, estão comumente expostas a situações que favorecem a ocorrência de acidentes de trabalho com exposição potencial a materiais biológicos, entre os quais agentes infecciosos que causam morte materna, como a infecção por HIV relatada por Tlou et al.^33^ com a *odds ratio* da mortalidade de 2,5, (IC95% 1,5–4,2). Outras doenças transmissíveis como o sarampo, rubéola, varicela, tuberculose, coqueluche, meningite, incluindo também as infecções causadas pelo vírus da influenza, citomegalovírus, VHB, VHC e parvovírus B19^25^ podem se associar indiretamente à morte materna. Outros fatores de riscos ocupacionais relevantes são as jornadas prolongadas, estresse emocional e físico, exposição a gases anestésicos e radiação^34^, associados a efeitos reprodutivos. A realização de parto por cesariana, que se associa a hemorragias, uma das causas mais comuns de morte materna, é mais frequente em enfermeiras que nas demais trabalhadoras^35^.

Entretanto, a maior parte dos estudos que trataram da análise de emprego ou ocupação e tipo de vínculo de trabalho não encontraram diferenças no risco de morte materna quando comparados a grupos de referência^5^, em concordância com os achados de Cecatti et al.^17^. As conclusões referentes às comparações desses resultados de estudos conduzidos em contextos bastante diversos – notadamente nos aspectos culturais, socioeconômicos e, consequentemente, do tipo de ocupação – são limitadas. Vale notar que grande parte desses estudos foram realizados com amostras muito pequenas e restrito poder estatístico.

Os achados do presente estudo mostram variações ocupacionais na razão de mortalidade materna, sugerindo que, além dos determinantes sociais já conhecidos, condições de trabalho podem ser informações importantes para a prevenção desse problema de saúde pública. Os dados do SIM têm escopo limitado por não permitirem análises que incluam fatores conhecidamente associados à morte materna, como o nível socioeconômico, condições de acesso e qualidade da atenção pré-natal, ou mesmo especificidades da ocupação, para ajuste ou estratificação. Nesse sentido, as conclusões devem ser consideradas com cautela. Dentre os limites metodológicos, é possível ter havido sub-registro de casos de morte materna. Esse problema foi demonstrado por Luizaga et al.^19^: ao estimar subenumeração de mortes maternas do SIM, que embora tenha se reduzido, ainda chegou a 21,4%. Um outro entrave metodológico relevante é a má qualidade e falta dos registros da ocupação no SIM e Sinasc, como foi documentado nos resultados deste estudo. Dados faltantes da ocupação foram mais comuns no SIM que no Sinasc, o que pode ser consequência da diferença de informantes relativa à fonte de dados, provocando assimetrias entre esses sistemas de informação. No SIM, a ocupação pode ser obtida de documentos da falecida, disponíveis para o atestante, ou de informação de alguém próximo, enquanto para o Sinasc a informante é a própria mãe. Além disso, a ocupação registrada pode não ser a verdadeira, considerando-se que as diferenças entre as múltiplas denominações e códigos da CBO limitam a real identificação e consequentemente o registro, o que remete à necessidade de melhor treinamento dos atestantes. Isso ficou evidente no grande número de registros inconsistentes, como a alta proporção de “donas de casa” (35,5%), que, embora seja uma ocupação empregada para fins previdenciários, não integra a CBO. No Brasil, a ocupação materna também deixou de ser registrada em 14,8% nas declarações de nascidos vivos^36^. Como é plausível a ocorrência desigual desses problemas de sub-registro de óbitos maternos ou a falta de dados sobre a ocupação entre regiões, unidades da federação ou entre as próprias ocupações, vieses podem ocorrer e distorcer os resultados nacionais apresentados. Uma outra possível origem de vieses é o uso de diferentes fontes, SIM e Sinasc, que podem ter diferenças operacionais no registro e fluxo de dados, embora ambas estejam abrigadas no Datasus e sujeitas a mecanismos de controle e implantação semelhantes. Ademais, a investigação de óbitos maternos vem avançando no país, ainda que de forma heterogênea; todavia, isso não vem ocorrendo no registro do campo ocupação.

A pesquisa sobre determinantes ocupacionais da morte materna necessita de investimentos, especialmente para estudos conduzidos com dados primários, detalhados, sobre as condições de trabalho e socioeconômicas, dentre outras. Os achados deste estudo podem contribuir para uma melhor visibilidade do problema e alertar para novos aportes voltados para grupos ocupacionais mais vulneráveis. Estratégias eficientes, nesse sentido, devem integrar ações judiciais e de fiscalização, regulamentações e a implementação de programas especiais, além de legislação destinada a garantir direitos humanos e trabalhistas.

## Footnotes

^a^ Ministério da Saúde (BR). Ministério da Saúde investe na redução da mortalidade materna. Brasília, DF: Agência Saúde; 28 maio 2018 [citado 21 dez 2018]. Disponível em: http://portalms.saude.gov.br/noticias/agencia-saude/43325-ministerio-da-saude-investe-na-reducao-da-mortalidade-materna
